# Enhancing Chlorobenzene Biodegradation by *Delftia tsuruhatensis* Using a Water-Silicone Oil Biphasic System

**DOI:** 10.3390/ijerph16091629

**Published:** 2019-05-10

**Authors:** Jie-Xu Ye, Tong-Hui Lin, Jing-Tao Hu, Rabin Poudel, Zhuo-Wei Cheng, Shi-Han Zhang, Jian-Meng Chen, Dong-Zhi Chen

**Affiliations:** 1 College of Environment, Zhejiang University of Technology, Hangzhou 310032, China; yejiexu@zjut.edu.cn (J.-X.Y.); cnlintonghui@163.com (T.-H.L.); jingtaoguyue@foxmail.com (J.-T.H.); Robin.pdl@yahoo.com (R.P.); zwcheng@zjut.edu.cn (Z.-W.C.); shihanzhang@zjut.edu.cn (S.-H.Z.); jchen@zjut.edu.cn (J.-M.C.); 2 Key Laboratory of Microbial Technology for Industrial Pollution Control of Zhejiang Province, Zhejiang University of Technology, Hangzhou 310014, China

**Keywords:** waste gas, monochlorobenzene, biodegradation, silicone oil, cell adhesion

## Abstract

In this study, a water–silicone oil biphasic system was developed to enhance the biodegradation of monochlorobenzene (CB) by *Delftia tsuruhatensis* LW26. Compared to the single phase, the biphasic system with a suitable silicone oil fraction (*v*/*v*) of 20% allowed a 2.5-fold increase in the maximum tolerated CB concentration. The CB inhibition on *D. tsuruhatensis* LW26 was reduced in the presence of silicone oil, and the electron transport system activity was maintained at high levels even under high CB stress. Adhesion of cells to the water–oil interface at the water side was observed using confocal laser scanning microscopy. Nearly 75% of cells accumulated on the interface, implying that another interfacial substrate uptake pathway prevailed besides that initiated by cells in the aqueous phase. The 8-fold increase in cell surface hydrophobicity upon the addition of 20% (*v/v*) silicone oil showed that silicone oil modified the surface characteristics of *D. tsuruhatensis* LW26. The protein/polysaccharide ratio of extracellular polymeric substances (EPS) from *D. tsuruhatensis* LW26 presented a 3-fold enhancement. These results suggested that silicone oil induced the increase in the protein content of EPS and rendered cells hydrophobic. The resulting hydrophobic cells could adhere on the water–oil interface, improving the mass transfer by direct CB uptake from silicone oil.

## 1. Introduction

Monochlorobenzene (CB) is a major volatile organic compound (VOC) emitted from the production of substances, such as nitrochlorobenzene, aniline, pesticides, adhesives, dyes, and drugs [1]. It has been listed as a priority pollutant by the American Environmental Protection Agency given its recalcitrance against removal and its biotoxicity and carcinogenicity [2]. Various techniques have been developed to remove CB from off-gas emissions, including biological methods [3], adsorption [4], photochemical methods [5], and catalytic oxidation [6,7]. Biological techniques are more attractive than physical and chemical methods because of their low cost, reliability, and environmental friendliness [8]. However, traditional biological treatments for hydrophobic and bio-refractory VOCs, such as CB, usually suffer from mass transfer limitations and microbial inhibition, which could lead to low elimination capacity and instability [9]. For instance, Wang et al. [10] observed that a prolonged recovery time was required after a transient loading operation, caused by the short-term increase in CB concentration when using a biofilter.

Some studies have suggested that the addition of an appropriate immiscible non-aqueous phase (NAP) could be a promising alternative to overcome the limitations of traditional biological techniques for hydrophobic VOC treatment [11,12]. Organic solvents, such as hexadecane, silicone oil, and hydrophobic ionic liquids, have been selected as NAPs because of their high affinity for target contaminants. The addition of NAPs has been successfully applied in the biological treatment of hydrophobic pollutants, such as hexane [13], styrene [14,15,16], toluene [17,18,19], dichloromethane [20,21], and α-pinene [22,23]. Nonetheless, there are comparatively few works concerned with their application in the biodegradation of CB.

The specific advantage of an aqueous-NAP system is the potential for self-regulation owing to the presence of NAP. The presence of NAPs can improve mass transfer of hydrophobic VOCs. Moreover, NAPs can absorb excess VOCs and deliver them at subinhibitory levels to microorganisms suspended in the aqueous phase [12,24]. Microbial activity, which is critical to biodegradation, could directly reflect the effect of NAP on substrate inhibition alleviation. However, it has rarely been considered in previous studies. Hence, whether NAPs can reduce microbial activity inhibition during CB degradation should be evaluated.

The substrate uptake pathway in the aqueous-NAP system would be affected by microbial characteristics because microorganisms with different hydrophobicities may be present in different phases [25]. Pollutants are commonly metabolized by microorganisms suspended in the aqueous phase [12,26,27]. Some researchers have stated that microbial growth and target compound removal occur at the aqueous-NAP interface [12,28,29]. Other researchers have even asserted that hydrophobic microorganisms could grow in the NAP and uptake the target substrate directly from the NAP [25,30]. Given that NAPs could modify microbial characteristics, such as cell hydrophobicity [31,32], detailed works on microbial distribution and cell hydrophobicity are required to explore the substrate uptake pathway involved in biodegradation of CB in an aqueous-NAP system.

*Delftia tsuruhatensis* LW26, which was successfully isolated and identified in our previous work [33], can utilize CB as its sole carbon and energy source. In this study, a silicone oil–water biphasic system was developed to enhance CB biodegradation by *D. tsuruhatensis* LW26, as silicone oil was the most commonly used NAP for its non-biodegradability and biocompatibility. CB abatement in the silicone oil–water biphasic system was investigated. The effect of silicone oil on the reduction in the inhibitory effects of CB on the microbial activity of *D. tsuruhatensis* LW26 was also evaluated. Furthermore, cell adhesion to the aqueous–oil interface and the surface characteristics of *D. tsuruhatensis* LW26 were evaluated in detail.

## 2. Materials and Methods

### 2.1. Chemicals

CB (99.0% purity), silicone oil (viscosity, 200 cSt at 25 °C), and 2-(*p*-iodophenyl)-3-(*p*-nitrophenyl)-5-phenyl tetrazolium chloride (INT) (98.0% purity) were purchased from Aladdin (Shanghai, China). All other chemicals used in this study were analytical grade (methanol was HPLC grade).

### 2.2. Microorganism and Culture Medium

*D. tsuruhatensis* LW26 (GenBank KP966097) was isolated from a lab-scale biotrickling filter reactor treating a CB-contaminated gas stream and was deposited at the China Center for Type Culture Collection (CCTCC M2011148, Wuhan, China). This strain can degrade CB and has a maximum specific growth rate of 0.42 h^−1^. It was cultivated at 30 °C (160 rpm) in a 300-mL sealed serum bottle containing 50 mL of sterilized mineral salt medium (MSM) with CB as the sole carbon and energy source. *D. tsuruhatensis* LW26 cells in the late exponential growth phase were harvested by centrifugation (8000 rpm, 10 min, 4 °C), washed twice, and then resuspended with MSM for further use as the fresh bacterial inoculum (OD_600_ = 0.2) in biodegradation experiments. MSM consisted of the following: 2.5 g/L (NH_4_)_2_SO_4_, 0.023 g/L CaCl_2_, 0.2 g/L MgSO_4_·7H_2_O, 1.0 g/L KH_2_PO_4_, 4.5 g/L Na_2_HPO_4_·12H_2_O, and 1 mL of trace element solution (pH 7.2). The trace element solution was prepared in accordance with Chen et al. [34].

### 2.3. Partition Coefficient Test

Partition coefficient tests were conducted in duplicate to assess the affinity of silicone oil for CB [22,35]. Sealed serum bottles were filled with 10 mL of silicone oil or water. A known amount of CB (2, 4, 8, 10, and 15 μL liquid CB) was then injected into the bottles, which were placed in a rotary shaker at 160 rpm for 24 h at 30 °C to establish the partition equilibrium. One milliliter of gas samples was withdrawn by using a gas-tight syringe and injected into a gas chromatograph (GC). The CB partition coefficient (*K*) was defined by Equation (1).

(1) $$K=C_{G}/C_{L}$$

where *C_G_* and *C_L_* represent the CB concentration in the gaseous and liquid phase, respectively. The coefficients were then obtained from the slope of the plot of the CB concentration in the liquid phase vs. the CB concentration in the gas phase.

### 2.4. Batch Biodegradation Experiments

Biodegradation of CB was performed in a biphasic system containing silicone oil and MSM. Three hundred-milliliter serum bottles were filled with 50 mL of sterile mixture of silicone oil and MSM, in which the silicone oil ratios were set to 10%, 20%, 35%, and 50% (*v/v*). A known amount of CB (10, 20, 30, 50 and 60 μL liquid CB, namely initial mass 11, 22, 33, 55, and 66 mg) was injected, and the flasks were inoculated with 1 mL of fresh bacterial inoculum (OD_600_ = 0.2). Finally, the sealed bottles were incubated under shaking condition (160 rpm) at 30 °C. The head-space samples were taken every 5 h, and subjected to GC analysis for the residual CB and the CO_2_ production. As a contrast experiment, CB was biodegraded following the same procedure in a single-phase system (in the absence of silicone oil). Furthermore, the abiotic serum bottles were conducted as blank control under the same conditions.

### 2.5. Electron Transport System Activity and Cell Surface Hydrophobicity Tests

Electron transport system (ETS) activity was measured by using a procedure similar to that reported by Trevors [36,37]. Cells were harvested by centrifugation (6000 rpm, 5 min, 4 °C) and washed twice with 10 mM phosphate buffer (pH 7.0). The washed cells were resuspended in phosphate buffer to obtain OD_600_ = 1.0. Next, 3 mL of the cell suspension and 100 μL of 2.0 mg/L INT solution were added into a glass test tube. The tube was placed in an incubator with shaking (100 rpm) at 30 °C for 30 min. Next, 10 μL of formaldehyde was added to stop the reaction. A subsample of cells harvested by centrifugation (6000 rpm, 5 min, 4 °C) was then extracted with HPLC-grade methanol. The mixture was mixed by using a vortex mixer for 1 min, and the methanolic extract was filtered through a preweighed Whatman No. 5 paper filter. The absorbance of INT-formazan in the methanolic extract was measured at 480 nm by using a Hitachi U-2910 spectrophotometer (Hitachi High Technologies, Tokyo, Japan) against a blank of a methanolic extract of cells minus INT. The extracted cells and filter were dried (105 °C, 24 h) to determine the dry weight of cells. ETS activity was expressed as μmol INT-formazan/(g-cell dry weight·h).

Cell surface hydrophobicity (CSH) was measured by using a test for bacterial adhesion to hydrocarbons (BATH) in accordance with the method described by Mishra and Singh [38]. The culture medium with cells was centrifuged at 6000 rpm for 5 min. The collected cells were then washed twice and resuspended in phosphate buffer to maintain an initial optical density at 600 nm of 0.6 ± 0.02. Four milliliters of bacterial suspension and 2 mL of hexane were added into a sterile test tube. Thereafter, the mixtures were mixed by vortex for 1 min and left standing for 30 min to achieve phase separation. Finally, the optical density of the obtained aqueous phase was measured again at 600 nm. CSH was calculated based on Equation (2).

(2) $$\mathrm{CSH}=\frac{{\mathrm{OD}}_{0}-{\mathrm{OD}}_{1}}{{\mathrm{OD}}_{0}}\times 100\%$$

where OD_0_ represents the initial OD_600_ and OD_1_ represents the OD_600_ after the CSH test.

### 2.6. Extracellular Polymeric Substance Analysis

Extracellular polymeric substances (EPS) were extracted from bacteria using the ultrasonic method [39,40]. Bacteria were harvested by centrifugation (6000 rpm) for 5 min at 4 °C, washed twice with 10 mM phosphate buffer (pH 7.0), resuspended with phosphate buffer to form a 15-mL suspension, and then ultrasonicated for 10 min at 20 kHz and 40 W. The suspension was centrifuged at 15,000 rpm and 4 °C for 20 min and was subsequently filtered through a 0.45-μm filter. The obtained EPS solution was stored at 4 °C before measurement. The protein in the EPS was quantified through the Lowry method with bovine serum albumin as a standard. The polysaccharide content in the EPS was determined by the phenol-sulfuric acid method with glucose as a standard.

### 2.7. Staining and Fluorescence Microscopy Observation

Cells were stained with blue fluorescent 4, 6-diamidino-2-phenyl-indole (DAPI, 10 μg/mL) in the absence of light at room temperature for 15 min. After that, cell pellets were collected, washed twice, and resuspended in PBS. Four milliliters of the DAPI-stained cells suspension were vigorously shaken with 1 mL of silicone oil, forming oil in water (o/w) emulsions. Spherical oil droplets from the o/w emulsion were transferred by using a micropipette to a slide glass and observed using confocal laser scanning microscopy (CLSM 780, Carl Zeiss, Oberlochen, Germany).

### 2.8. Analytical Methods

CB concentration was analyzed by a GC (6890N, Agilent Technologies, Palo Alto, CA, USA) coupled with a flame ionization detector and a HP-Innowax capillary column (30 m × 0.32 mm × 0.5 μm). The temperatures of the injector, column and detector were maintained at 200 °C, 100 °C and 180 °C, respectively. The carrier gas was N_2_, at a flow rate of 33.4 mL/min.

CO_2_ concentration was measured on a GC (6890N, Agilent Technologies) equipped with a thermal conductivity detector and an HP-Plot-Q capillary column (30 m × 0.32 mm × 20 μm). The temperatures of the injector, column and detector were 90 °C, 40 °C, and 180 °C, respectively. Helium was used as a carrier gas at a rate of 5 mL/min.

Cell concentration was determined by optical density using a spectrophotometer (U-2910, Hitachi High Technologies, Tokyo, Japan) at 600 nm. A calibration curve between cell concentration and optical density was constructed prior to this analysis (*y* = 222.93*x* − 0.69, where *y* = mg/L cell concentration and *x* = OD_600_).

## 3. Results

### 3.1. Affinity of Silicone Oil for CB

The affinity of silicone oil for CB was assessed based on the partition coefficients. The air/oil and air/water partition coefficients were estimated to be 0.00066 and 0.24, respectively. CB was approximately 350 times more soluble in silicone oil than in water. The air/oil partition coefficient obtained in this work was comparable with that obtained by Muñoz et al. [22], who reported that the partition coefficient of α-pinene in silicone oil was 0.000183, and was considerably lower than the partition coefficient of hexane (0.0034) reported by Arriaga et al. [35]. In addition to its great affinity for CB, silicone oil was demonstrated to be biocompatible with *D. tsuruhatensis* LW26 and non-biodegradable by *D. tsuruhatensis* LW26 under the tested experimental conditions (data not shown). Thus, silicone oil was considered to be an appropriate NAP in the biphasic system for trapping CB from waste gas.

### 3.2. Performance of the Water–Silicone Oil System

**Figure 1:**
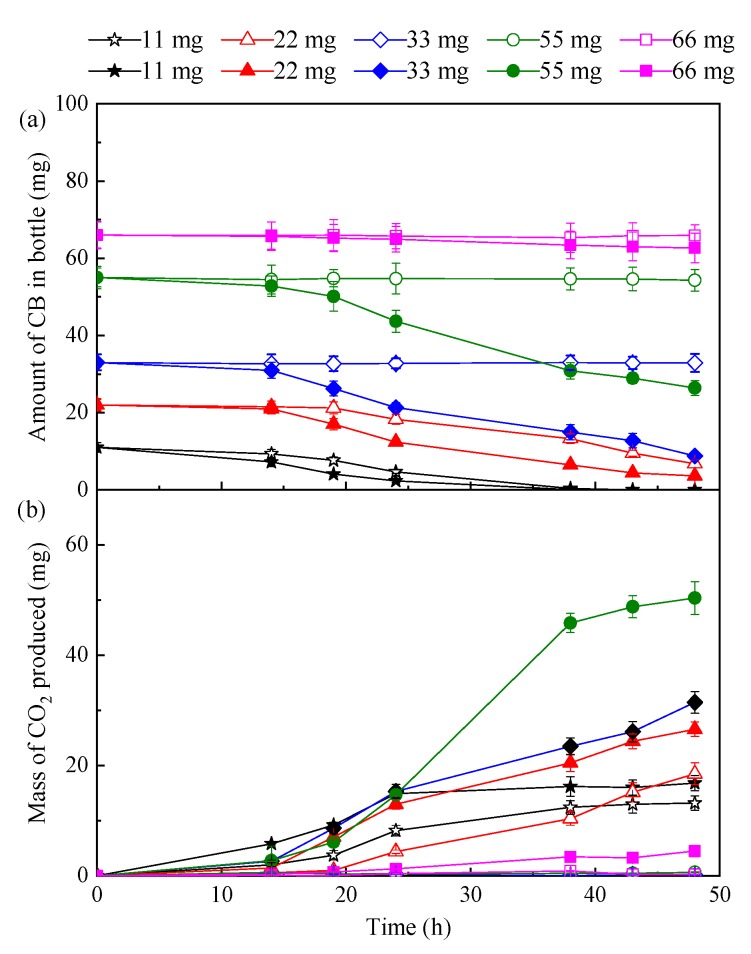
(**a**) Monochlorobenzene (CB) biodegradation and (**b**) CO_2_ production profiles of the biphasic system (20% silicone oil, solid symbols) and single-phase system (empty symbols) under different CB dosages.

CB degradation experiments in the water–silicone oil biphasic system (20%) were conducted with various CB dosages to determine the CB degradation ability of *D. tsuruhatensis* LW26 in the presence of silicone oil. Degradation experiments without silicone oil were also performed. As shown in Figure 1a, CB was almost completely degraded in the presence of 11 mg of CB after 38 h of incubation. Therefore, CB biodegradation by *D. tsuruhatensis* LW26 was faster than that by some other microorganisms, such as *Ralstonia pickettii* L2 [41] and fungal–bacterial consortium [3]. For example, the fungal–bacterial consortium required approximately 60 h to degrade 10 mg of CB. Furthermore, from Figure 1a it is evident that CB biodegradation in the biphasic system was superior to that in the single-phase system. When 22 mg CB was added to the test bottle, the degradation efficiency of the single-phase system was 69.3% after 48 h of incubation; however, as the CB dosage increased to 33 mg, no obvious CB removal could be found, suggesting a maximum substrate tolerance of 22 mg CB. By contrast, 83.8% of 22 mg CB was degraded in the presence of silicone oil within 48 h, and the maximum substrate tolerance achieved in the biphasic system was 55 mg CB, corresponding to a 2.5-fold enhancement compared to results obtained in the single-phase system. The high substrate tolerance of *D. tsuruhatensis* LW26 in the biphasic system could mainly be attributed to the regulatory effect of silicone oil. The presence of silicone oil was able to reduce the concentration of CB in the aqueous phase. Therefore, the metabolic activity of LW26 was speculated to remain high. CO_2_ generation profiles were also monitored, given that CO_2_ production could be used as an indicator of biodegradation and mineralization [18,24]. As depicted in Figure 1b, the increment in the mass of the produced CO_2_ provided valuable evidence for CB mineralization by *D. tsuruhatensis* LW26. The higher CO_2_ production rates of the biphasic system than those of the single-phase system for CB biodegradation confirm the positive effect of silicone oil. 

**Figure 2:**
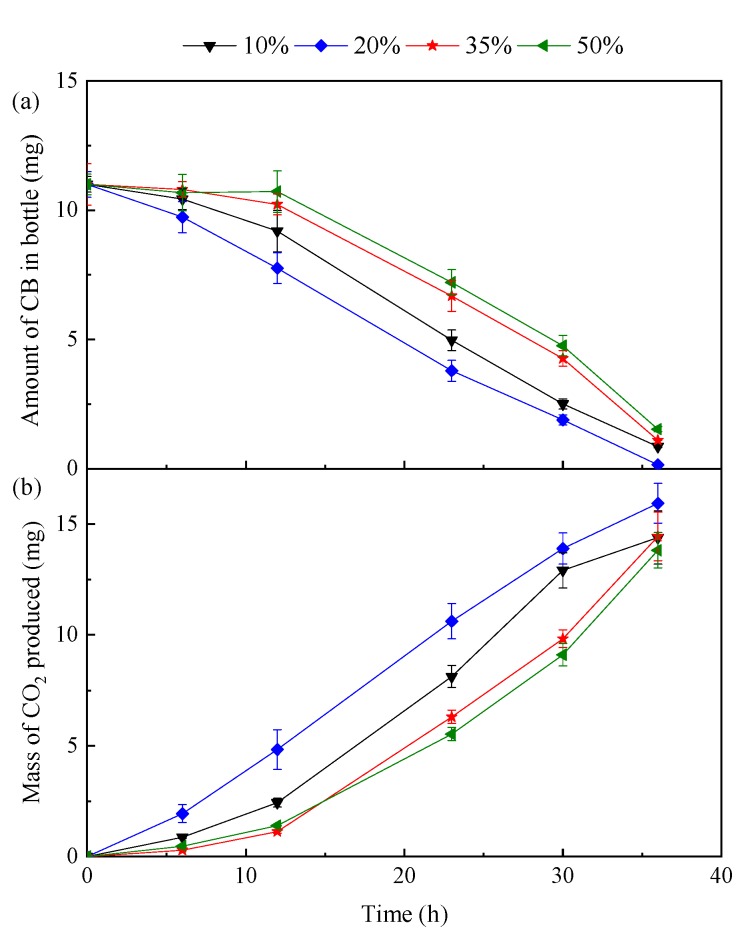
(**a**) CB biodegradation and (**b**) CO_2_ production profiles of the biphasic system with different volume fractions of silicone oil. The initial CB addition was 11 mg in each test bottle.

CB biodegradation by *D. tsuruhatensis* LW26 as a function of the volume fraction of silicone oil was also investigated. As illustrated in Figure 2a, the CB removal efficiency was best when using 20% silicone oil. For example, 65.5% of CB was removed within 23 h in the case of 20% silicone oil, while the removal efficiencies of CB in the presence of 10%, 35%, and 50% silicone oil were only 54.8%, 39.3%, and 34.5%, respectively. Moreover, the average CB removal rates for volume fractions of 10%, 20%, 35%, and 50% were 0.28, 0.30, 0.27, and 0.26 mg/h, respectively, after 36 h of incubation. It was observed that the variation of CO_2_ production rates with the increasing silicone oil fraction followed a similar trend as that of CB removal rates (Figure 2b). Thus, the silicone oil fraction influenced the removal of CB and the optimal silicone oil fraction was 20%. Clarke and Correia [42] reported that the NAP fraction has a great effect on the overall mass transfer coefficient (*K*_L_*a*). *K*_L_*a* can either increase continuously with increasing NAP fraction or peak at a certain critical NAP concentration. Therefore, the optimal silicone oil fraction of 20% in the present study could be likely explained by high CB availability due to an optimal *K*_L_*a*.

### 3.3. ETS Activity Assays

ETS activity is the impetus of numerous complex metabolic processes, including the catabolic oxidation between organic matter and O_2_ [43,44]. The ETS activities of *D. tsuruhatensis* LW26 in the biphasic and single-phase systems were evaluated to further understand the function of silicone oil in protecting *D. tsuruhatensis* LW26 against CB toxicity. As shown in Table 1, ETS activities in the single-phase system (384.2 ± 51 μmol-INTF/(g cell dry weight·h)) and the biphasic system (396.4 ± 25 μmol-INTF/(g cell dry weight·h)) were almost equal when 11 mg of CB were added, which meant that the biodegradation activity was not inhibited in this condition. Therefore, the higher CB removal rate in the biphasic system under the CB dosage of 11 mg (Figure 1) indicated that silicone oil addition could improve mass transfer.

Furthermore, the results presented in Table 1 showed that ETS activity in the single-phase system dropped dramatically with increasing initial CB concentration, and it was not detected when 33 mg CB was added to the test bottle. This suggested that biodegradation in the single-phase system would be completely inhibited under CB dosages of more than 33 mg. However, ETS activity in the biphasic system could still be maintained at 234.9 ± 27 μmol-INTF/(g cell dry weight·h) in the case of a CB dosage of 33 mg. These results demonstrated that the superior performance obtained in the water–silicone oil system at high CB concentrations (Figure 1) resulted from the protective effect of silicone oil on microorganisms against substrate inhibition. Other studies have identified similar benefits for the biodegradation of VOCs with the addition of silicone oil and silicone rubber [20,45].

### 3.4. Microbial Distribution in the Water–Silicone Oil System

Silicone oil in the biphasic system dispersed into numerous spherical droplets during shaking in batch biodegradation experiments. The mixture inoculated with *D. tsuruhatensis* LW26 separated into an o/w emulsion layer and water layer after 1 h of gravity separation, and the o/w emulsion remained stable for days without noticeable signs of destabilization. By contrast, oil droplets coalesced immediately into distinct oil and water layers in the control experiment without bacterial cells. The stability of visible oil droplets in the biphasic system implied that bacterial cells might assemble at the surfaces of oil droplets and hinder the coalescence of oil droplets.

**Figure 3:**
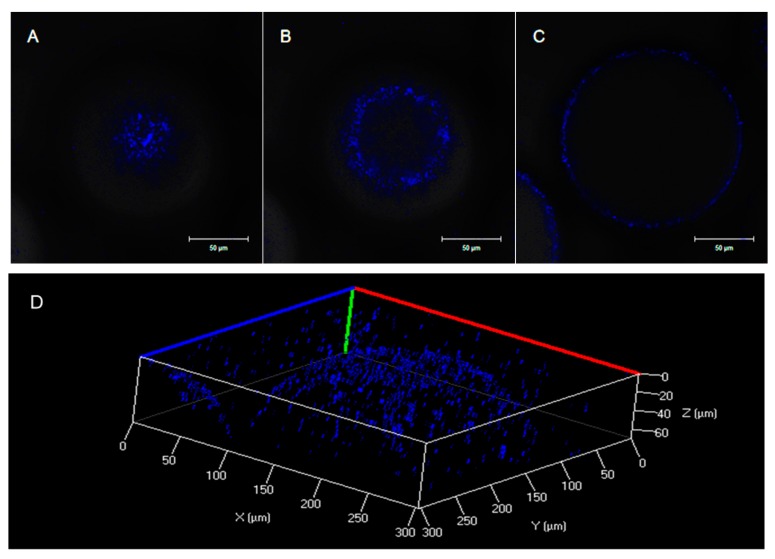
Confocal images of emulsion stabilized by *D. tsuruhatensis* LW26 stained with fluorescent 4, 6-diamidino-2-phenyl-indole (DAPI). Bacterial cells mainly localized at the interface of silicone oil droplets in the blue fluorescent field at z = (**A**) 5, (**B**) 15, and (**C**) 45 μm. Scale bars represent 50 μm. (**D**) Microbial distribution in the 3D system.

To confirm that *D. tsuruhatensis* LW26 cells adhered to the water–silicone oil interface, a DAPI-stained cell suspension was mixed with silicone oil (20%) and then analyzed by CLSM under a blue fluorescent field. The cross sections of the droplets at z = 5, 15, and 45 μm are shown in Figure 3A–C. The circular blue fluorescence only appeared at the perimeter of the oil droplet, while the inside remained dark, which means DAPI-stained cells existed at the surface of the oil droplets and formed a thin biofilm. Meanwhile, the microbial distribution in the 3D system, shown in Figure 3D, intuitively illustrated that numerous bacterial cells were located at the water–oil interface and had grown at the water side of the interface. The phenomenon depicted above suggested that CB uptake not only happened in the aqueous phase, but also might occur at the water–oil interface. Notably, when adding 11 mg of CB, the CB concentration in the single-phase system was measured to be 95.2 mg/L on the basis of the thermodynamic equilibrium between the gas/NAP/aqueous phases and was 30 times greater than that in the aqueous phase of the biphasic system (2.9 mg/L). Moreover, the activities of *D. tsuruhatensis* LW26 were not inhibited (Table 1). The degradation rate of the former system at the CB dosage of 11 mg should be higher than that of the latter if CB uptake only occurs in the aqueous phase. However, the contradictory trend shown by the results displayed in Figure 1 indicated that CB uptake in the aqueous phase is not the dominant pathway. Therefore, it can be proposed that the interfacial substrate uptake by the bacterial cells on the water–oil interface contributed to CB degradation. Cells at the interface came into direct contact with silicone oil and acquired CB by transfer near the point of contact through their membranes. Macleod and Daugulis [46] also elucidated the importance of the interfacial uptake pathway but found that organisms adhered on the NAP side of the aqueous-bis(ethyl hexyl) sebacate (BES) interface. The above difference might be attributed to the different microorganisms and NAPs employed in the biphasic system.

**Figure 4:**
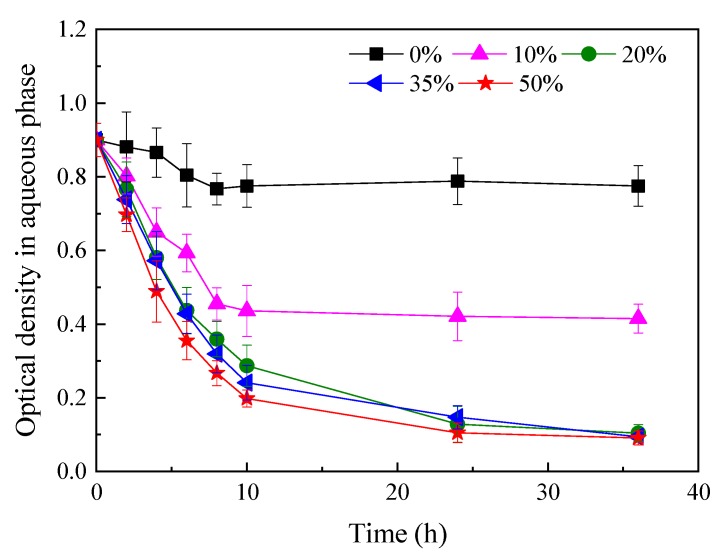
Time course of the variation in optical density in the aqueous phase of the biphasic system. Serum bottles (300-mL) were filled with 50 mL of a sterile mixture of silicone oil and mineral salt medium (MSM) in the absence of CB. Different volume fractions of silicone oil (0–50%) were used.

The distribution of bacterial cells in the biphasic system with different silicone oil fractions was further investigated. The profiles in Figure 4 revealed that the optical density of the aqueous phase in the biphasic systems in the presence of all silicone oil fractions sharply decreased during the initial 10 h of the experiment and then gradually stabilized. This result should be ascribed to the gradual adsorption of bacterial cells to the water–oil interface until the interface reached saturation. NAP proportion had a considerable impact on biomass distribution between water and oil (Figure 4). Bacterial adhesion to the water–oil interface intensified as the silicone oil fraction increased from 0% to 20%. The optical density of the aqueous phase decreased to 0.1 in the presence of 20% silicone oil. Specifically, 75% of cells were absorbed. However, further increasing the silicone oil fraction to 50% did not result in a corresponding increase in microbial absorption. These results suggest that the expansion of the interfacial area of the biphasic system with a silicone oil fraction of >20% (*v/v*) might be limited. Interfacial area is critical for substrate transfer and uptake in the biphasic system, especially in the presence of the interfacial substrate uptake. The aforementioned result confirmed that the maximum CB degradation rate at a silicone oil/water ratio of 20% (Figure 2) might be attributed to the optimal interface area. A higher interfacial area led to an increase in the microbial adhesion at the water–silicone oil interface, which improved mass transfer and enhanced the CB degradation rate.

### 3.5. Effect of Silicone Oil on the Surface Characteristics of D. tsuruhatensis LW26

**Figure 5:**
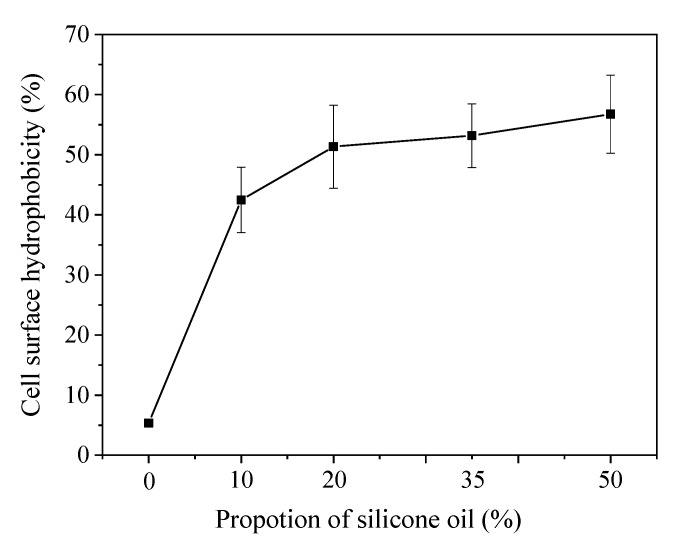
Effects of silicone oil on the cell surface hydrophobicity (CSH) of *D. tsuruhatensis* LW26.

Bacterial cell adhesion to the water–oil interface should be related to the cell surface characteristics of *D. tsuruhatensis* LW26. CSH is an inducible cell property. Neumann et al. [31] found that the CSH of *Pseudomonas putida* DOT-T1E increased by 3-fold in the presence of 10% (*v/v*) 1-decanol. However, a hydrophobicity shift is difficult to predict given that the effects of NAP on the hydrophobic surface properties differ greatly; it is usually dependent on bacterial species, cultivation mode, and NAP types [25,32,47]. Thus, the influence of silicone oil on the CSH of *D. tsuruhatensis* LW26 was estimated. The results depicted in Figure 5 indicated that the silicone oil addition to 20% resulted in an increase of the CSH of *D. tsuruhatensis* LW26. CSH reached a value of 51 ± 7% at an oil fraction of 20%, attaining a 10-fold increase in comparison with that in the single-phase system. The further increase in silicone oil addition from 20% to 50% had an insignificant impact (*p* = 0.439, Kruskal–Wallis test) on the CSH. As *D. tsuruhatensis* LW26 turned hydrophobic with the addition of silicone oil, cells in the biphasic system tended to form a biofilm at the water–oil interface, and the uptake of CB by contact between cells and hydrophobic CB molecules was facilitated. This finding was different from the results reported by Hernández and Muñoz Torre [32], in which no evident CSH shift was observed in *Pseudomonas aeruginosa* in the presence of silicone oil. The differences might be mainly attributed to the different microorganism used. 

EPS has been suggested to be correlated with cell hydrophobicity because it contains hydrophobic proteins and hydrophilic polysaccharides [48]. EPS contents were identified in order to further understand the increase in CSH in the presence of silicone oil. As shown in Table 2, the EPS and CSH values in the biphasic system were 41.7 ± 8 mg/g and 42.5 ± 5.4 mg/g, a 1.4-fold and 8-fold improvement compared with that in the single-phase system, respectively. Furthermore, the extracellular protein/polysaccharide (PN/PS) ratio was 4.6 in the biphasic system, 3-fold higher than that obtained in the single-phase system. This result indicated that the enhancement in CSH observed in the present study might depend on the increased protein content and decreased polysaccharide content of EPS. Therefore, silicone oil induced the variation in EPS constituents and modified the cell hydrophobicity. The resulting hydrophobic microbial cells then adhered on the water–oil interface, improving the CB uptake rates.

## 4. Conclusions

This study confirmed that the water–silicone oil biphasic system can drastically improve the performance of CB degradation by *D. tsuruhatensis* LW26 and that the optimal volume fraction of silicone oil was 20%. The maximum tolerated CB concentration of the biphasic system increased by 2.5-fold relative to that of the single-phase system. The biphasic system reduced the inhibitory effect of CB on *D. tsuruhatensis* LW26 and maintained ETS activities at high levels. In addition, most of the bacterial cells accumulated on the water–oil interface, mainly due to the increase in cell hydrophobicity in the presence of silicone oil. The results of this study indicated that the interfacial uptake pathway was prevalent in the waster–silicone oil system, and thus promoted the mass transfer and CB biodegradation. These findings could facilitate the optimization of two-phase partitioning bioreactors for treating CB-contaminated waste gas. Further systematic pilot-scale studies should be performed before full-scale application.

## Figures and Tables

**Table 1:** Electron transport system (ETS) activity in the single-phase and biphasic systems (20% silicone oil).

Initial Amount of CB in Serum Bottle (mg)	ETS Activity (μmol-INTF/(g Cell Dry Weight·h))	
	Single-phase system	Biphasic system
11	384.2 ± 51	396.4 ± 25
22	182.7 ± 25	283.5 ± 32
33	--	234.9 ± 27

**Table 2:** CSH and EPS in the single-phase and biphasic systems (20% silicone oil).

System	CSH (%)	Protein in EPS (mg/g)	Polysaccharide in EPS (mg/g)	EPS (mg/g)	PN ^a^/PS ^b^
Single-phase system	5.3 ± 0.4	18.2 ± 3.5	12.3 ± 3.1	30.5 ± 6.6	1.5
Biphasic system	42.5 ± 5.4	34.2 ± 5.8	7.5 ± 2.2	41.7 ± 8	4.6

EPS: extracellular polymeric substance; ^a^ PN: the protein in EPS. ^b^ PS: the polysaccharide in EPS.
